# 

**Published:** 1896-08

**Authors:** 


### CONTENTS.

COMMUNICATIONS.

Antiseptics .......... 365
Physiological Antagonism .......... 369
Address of Dr. W. W. Shyrock, President of the Indiana State Dental Association, at Indianapolis, June 30th, 1896 .......... 373
Necrosis .......... 375
What Should the Dentist do to Prevent Decay of the Teeth .......... 379
The Absorption of the Roots of the Temporary Teeth .......... 388
Then and Now .......... 396
Benefits of Dental Society Meetings .......... 404
The Dentist .......... 406

COMMENCEMENTS.

Detroit College of Medicine .......... 410
Richmond Univers ty College of Medicine .......... 411
Western Reserve University—Dental Department .......... 412
Toronto Royal College of Dental Surgeons .......... 412

SELECTIONS.

The American Medical Association .......... 405
Taking the Air without going out .......... 416

EDITORIAL.

Cincinnati Academy of Dentistry .......... 413
Biographical - Dr. P. G. C. Hunt .......... 413

